# Photobehaviours guided by simple photoreceptor systems

**DOI:** 10.1007/s10071-023-01818-6

**Published:** 2023-08-31

**Authors:** Emelie Brodrick, Gáspár Jékely

**Affiliations:** 1 Living Systems Institute, University of Exeter, Stocker Road, Exeter, EX4 4QD UK; 2 Centre for Organismal Studies, University of Heidelberg, 69120 Heidelberg, Germany

**Keywords:** Phototaxis, Non-visual photoreception, Photokinesis, Photophobia, Scotophobia

## Abstract

Light provides a widely abundant energy source and valuable sensory cue in nature. Most animals exposed to light have photoreceptor cells and in addition to eyes, there are many extraocular strategies for light sensing. Here, we review how these simpler forms of detecting light can mediate rapid behavioural responses in animals. Examples of these behaviours include photophobic (light avoidance) or scotophobic (shadow) responses, photokinesis, phototaxis and wavelength discrimination. We review the cells and response mechanisms in these forms of elementary light detection, focusing on aquatic invertebrates with some protist and terrestrial examples to illustrate the general principles. Light cues can be used very efficiently by these simple photosensitive systems to effectively guide animal behaviours without investment in complex and energetically expensive visual structures.

## Introduction

In any habitat that natural light reaches, the majority of organisms, from vertebrates to bacteria, make use of it in some way, either for photosynthetic autotrophy or for learning about their surroundings. Many animals that live in dark places, such as caves or deep water, even make their own light via bioluminescence (Kricka 2005). Light provides both a vital energy source and a cue for orientation and navigation, hunting and communication. However, sunlight can also be dangerous, directly by its electromagnetic radiation, or indirectly, by its power to expose an individual to the eyes of another. There is a huge diversity of light-sensing strategies since photoreception and visual systems arose in animals early in evolution and have since been lost, re-invented and improved many times in many ways (Land and Nilsson 2012; Picciani et al. 2018). In this review, we explore the photoreceptor cells and photobehaviours of animals that detect light but do not “see” using spatial vision and image-forming eyes. How do these organisms use simple forms of photoreception and optical mechanisms to guide effective behaviours to navigate and survive a world that many others around them can see well? We will summarize the diversity of photoreceptor cells, visual pigments and optical structures present across animals and provide an overview of rapid behavioural response mechanisms to light. We also propose some robust experimental designs to meaningfully categorize and quantify the responses.

### Photoreceptor cells

We consider a photoreceptor cell to be any cell expressing photoexcitory proteins in the plasma membrane that can initiate a phototransduction cascade when exposed to light. Photoreceptors can exhibit very distinctive morphology such as expansive cell membranes as they adapted to enhance light-gathering power by absorbing more photons across a greater surface area. Classically, these cell types were separated into two distinct groups based on their morphological characteristics (Eakin 1963, 1965). Ciliary photoreceptor cells have ciliary projections on the plasma membrane and hyperpolarize on phototransduction. These are found, for example, in vertebrate-eye rod and cone cells. Rhabdomeric photoreceptor cells bear microvilli, produce depolarizing signals and are found in arthropod compound eyes and many other invertebrate taxa. Contrary to early ideas, both of these major cell types can be found in both vertebrates and invertebrates (Porter et al. 2012). There are also other photoreceptor types that do not fit into either of these two groups. An unusual cell type found in larval chitons bears both cilia and microvilli (Vöcking et al. 2017). Photoreceptor cells can also be inconspicuous in appearance, lacking copious membrane elaborations, making them difficult to identify with an electron microscope. Furthermore, they are commonly located outside eyes (extraocular) and there are examples of photoreceptors in almost every animal tissue type, from the skin of sharks (Delroisse et al. 2018) and cephalopods (Kingston et al. 2015) to deep within the dark human brain (Blackshaw and Snyder 1999; Halford et al. 2001).

### Visual pigments

Light sensing is commonly mediated by opsins and any one animal can express a surprisingly high number of different opsins, used in eyes and various other parts of the body (Terakita 2005; Leung and Montell 2017; Lowe et al. 2018; Macias-Muñoz et al. 2019). Opsins are G-protein-coupled receptors, consisting of seven transmembrane domains arranged in a ring. They form a photopigment when bonded to a central vitamin A-based retinaldehyde chromophore, and when excited by light initiate a phototransduction cascade in the photoreceptor cell (Shichida and Matsuyama 2009; Hardie and Juusola 2015).

Typically, ciliary photoreceptor cells express c-opsins, while rhabdomeric photoreceptors express r-opsins in the membrane. However, there are many other opsin classes with cases of co-expression in a cell. The aforementioned chiton larva has photoreceptors with both cilia and microvilli (Vöcking et al. 2017), which express both r-opsin and xenopsin as a visual pigment (Ramirez et al. 2016; Rawlinson et al. 2019; Döring et al. 2020). Despite the relatively recent recognition of xenopsin, it is very prevalent among protostome invertebrates (Wollesen et al. 2023), but appears to have been frequently lost and is not present outside lophotrochozoans (Arendt 2017). There are several other unique classes of opsins too (Ramirez et al. 2016), such as the tetraopsins (Porter et al. 2012) and Cnidaria-specific cnidopsins (Bielecki et al. 2014; Liegertová et al. 2015), to name just two. Though numerous and prevalent, these other opsin classes have only begun to receive attention in recent years. Opsins are very diverse and have a complicated classification, not discussed in detail here [for opsin phylogeny see (Ramirez et al. 2016; Gühmann et al. 2022)]. They are ancient proteins that emerged before the last common ancestor of bilaterians (Ramirez et al. 2016). Therefore, bilaterians have inherited representatives from more than one class of opsin. Across many invertebrates there are cases where an animal still possesses both the ancestral ciliary and rhabdomeric opsins and associated cell types. In these organisms the two types can be incorporated into different structures (McReynolds and Gorman 1970; Eakin and Brandenburger 1981; Arendt et al. 2004; Vopalensky et al. 2012) or may even coexist simultaneously in the same organ. For example, the two retinal layers of the scallop eye each contain a different type of opsin and photoreceptor cell. The distal retina contains hyperpolarizing ciliary photoreceptors that contain G_o_-opsin (belonging to a distinct opsin class), whereas, the proximal retina has more sensitive rhabdomeric photoreceptor cells that depolarize in response to light and express an r-opsin (McReynolds and Gorman 1970; Kojima et al. 1997).

There are other photosensitive protein families that can mediate extraocular light sensing (see (Porter 2016) for a review). One other major and widespread family of photosensory molecules that deserves mention is the cryptochromes. These photolyase-like flavoproteins are sensitive to short-wavelength (UV-A/blue) light (Lin and Todo 2005; Chaves et al. 2011; Lopez et al. 2021), but they are not considered to be true visual pigments. Among invertebrates, they are involved in a wide variety of light-sensing functions, including entrainment of circadian clocks (Cashmore 2003; Michael et al. 2017; Damulewicz and Mazzotta 2020) and moon cycles for synchronization of spawning events (Levy et al. 2007; Poehn et al. 2022) and magnetoreception in flying insects (Gegear et al. 2008; Bazalova et al. 2016; Wan et al. 2021; Netušil et al. 2021). Sponge larvae, which lack any opsins, provide a rare case of an animal proposed to use cryptochromes to facilitate photobehaviour via sensing spatial aspects of the light environment (Rivera et al. 2012).

### Non-visual photoreceptors and eyes

We will discuss details on a diverse range of animals and light-sensing apparatus, so we will first clarify the terminology on different cell types and organs, using some of the distinctions made by Cronin and Johnsen (Cronin and Johnsen 2016). A non-visual photoreceptor is any cell of any body region that is photosensitive, but does not contribute to vision or sensing spatial aspects of light.

Some of these non-visual photoreceptors are actually found inside eyes (ocular), but rather than providing visual information to the nervous system, they are used to sense light for accessory functions, such as pupillary modulation by the iris or photo-entrainment in mammals (Van Gelder et al. 2003; Hattar et al. 2003; Margiotta and Howard 2020). In this case, a non-visual population of ganglion cells, the intrinsically photosensitive retinal ganglion cells (ipRGCs), found sparsely in the ganglion cell layer of the retina, mediate the responses. The non-visual light-sensing functions of the ipRGCs are mediated by melanopsin, a member of the r-opsin family (Hattar et al. 2003; Sexton et al. 2012). Some non-visual photoreceptors can be found in other parts of a body (extraocular), for instance the dermal photoreceptors that mediate dynamic adaptive body colour changes in animals such as peppered moths (Eacock et al. 2019) and cephalopods (Kingston et al. 2015; Al-Soudy et al. 2021). These are non-visual photoreceptors but their placement outside of eyes means they can be further termed extraocular photoreceptors.

We consider complex eyes to be organs capable of sensing aspects of space and motion using optical components to direct light onto a spatially matched retinal array of photoreceptors. Complex eyes include compound and camera eyes and also other types of complex optical solutions (e.g. parabolic mirror eyes). Upon phototransduction, signals with light information are usually transmitted neurally to the central nervous system to form an image. Simple eyes, also called by other names, including “eye spots”, “pit eyes” and “ocelli”, are present in a wide variety of invertebrates. They can exist singly or in various numbers to provide the primary visual apparatus for an animal. Alternatively, they may be found in conjunction with a set of larger image-forming complex eyes. There are many designs of simple eyes, some concave in shape, some convex, and there is great variation in the optical components. In fact, the only unifying aspect of this catch-all term is that they are small in size and relatively simple in anatomy. While simple eyes do not usually collect highly resolved light information, they can often be used to sense light direction, for example by having pigments that shade part of a photoreceptor array. There are, of course, optical structures that lie somewhere between a simple eye and a complex eye, such as the dozens of small round scallop eyes, which look out of the shell from the mantle’s edge. These eyes are exceptional in their resolving power and design, each possessing a lens, pupil and concave parabolic guanine mirror that reflects light towards two layers of retina (Land 1965; Palmer et al. 2017).

Complex animal eyes have been the subject of scrutinous research and extensive review over the years (Arendt 2003, 2008; Nilsson and Arendt 2008; Arendt et al. 2009; Nilsson 2009, 2013; Land and Nilsson 2012; Oakley and Speiser 2015; Randel and Jékely 2016). However, extaocular and simple forms of photoreception have received less attention despite being extremely widespread and prevalent across animal lineages. We will classify and review non-visual photoreception strategies employed to guide locomotive responses. These behaviours can be categorized into four main types: photophobia, scotophobia, photokinesis and phototaxis (Diehn et al. 1977; Nultsch and Häder 1988; Wilde and Mullineaux 2017), and each allows animals to navigate their environment and escape predators, avoid harmful sunlight and maintain position within a preferred environment. We explore how animals achieve and guide these behaviours without complex eyes.

## Non-visual photobehaviour

### Photophobia

Photophobia (light avoidance) is a behavioural aversion to an increase in light intensity (Diehn et al. 1977; Nultsch and Häder 1988). When exposed to brighter light, a photophobic animal may respond, often immediately, by a muscular body contraction, burst of movement or the arrest of ciliary beating. They might freeze, change their movement velocity or turn to change direction. One major driver for photophobia among zooplankton in open water is the risk of exposure to visual predators (Brierley 2014). An animal swimming towards the water surface may change its direction or movement velocity upon detection of light intensities above a certain threshold. This would prevent travel further into bright light and allow the animal to sink or swim down again into deeper water where there is lower predation pressure and light exposure. Photophobic responses can thus remove an individual from an undesirably bright environment without the need for image-forming eyes or even directional photoreception.

**Fig. 1:**
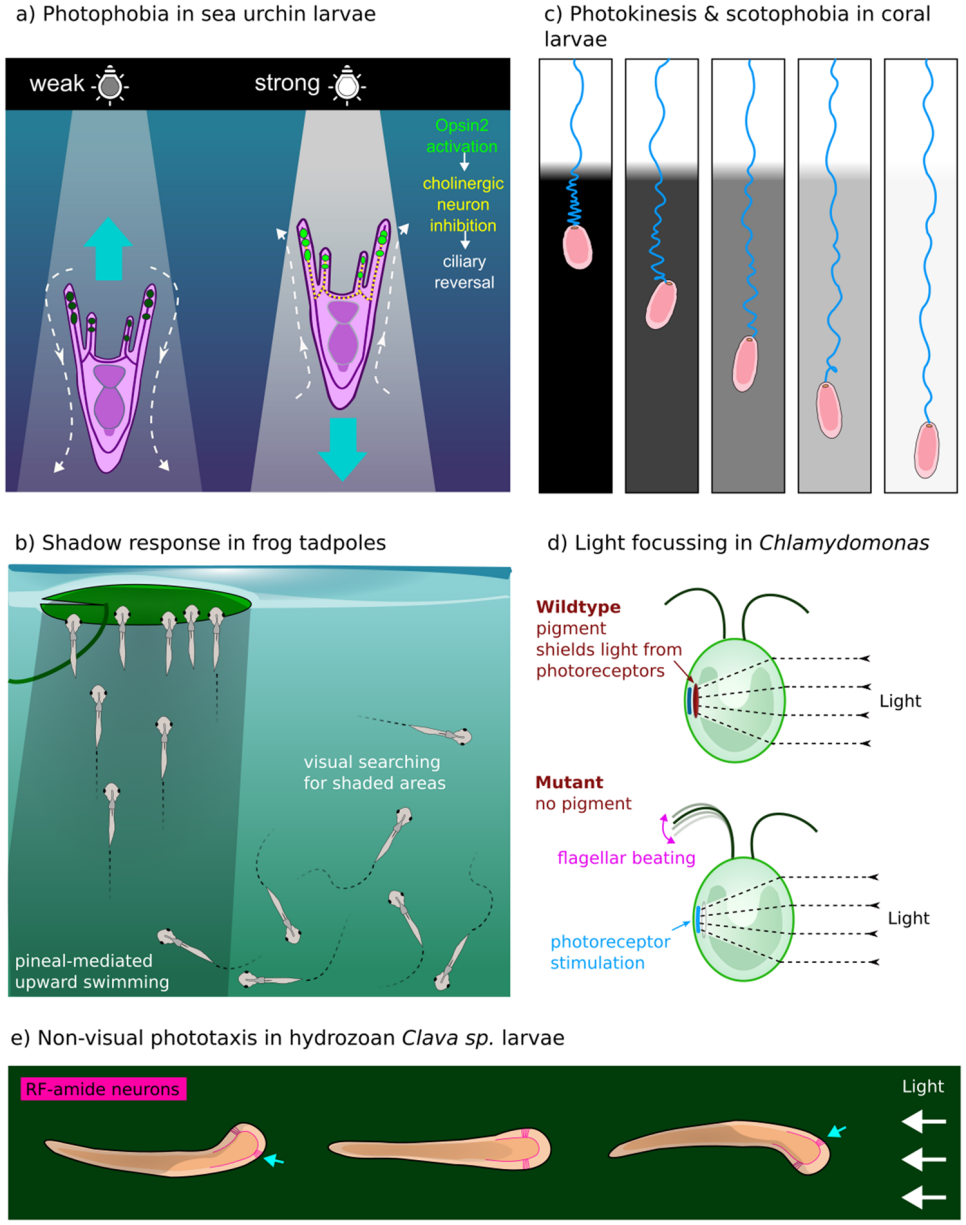
**a** Photophobia in sea urchin larvae. Bright light induces reversal of swimming direction via Opsin2 activation in arm mesenchymal cells. This inhibits the cholinergic neural signalling that regulates forward ciliary beating (Yaguchi et al. 2022). Larvae entering above-threshold levels of strongly illuminated surface waters will switch to backward swimming into deeper water. White dotted arrows indicate flow direction of the cilia and large blue arrows show the larva’s direction of travel. **b** Xenopus frog tadpoles swim actively under bright light, but when entering a darkened region such as the shadow of a lily pad, the pineal eye elicits an upward swimming response in the tadpole (Jamieson and Roberts 2000). This allows it to locate shaded vegetation on the surface, on which it prefers to attach. **c** Acroporid coral larvae swim actively in bright light. Hypothetical trajectories travelled over a fixed time period are represented by the blue lines behind the larvae. On sudden experiencing a sudden dimming of light intensity, they show a scotophobic response by slowing forward movement (Sakai et al. 2020). The average speed change is proportional to the intensity change, suggesting a photokinetic response mechanism. **d** Mutants lacking eyespots in the unicellular alga *Chlamydomonas reinhardtii* show a reversed phototactic response to light stimulation, relative to wild types that possess eyespots (Ueki et al. 2016). They are able to achieve this because the convex shape of the cell itself focuses light onto the cell wall photoreceptors region opposite from the light source, creating a focused area of stimulation. **e** The planula larva of the hydrozoan *Clava multicornis* swings its aboral front end (“head”) as it crawls along macroalgae substrates away from its parent. The “head” has two loose aggregations of RF-amide-expressing neurons (pink), which appear to be involved in intensity signal comparisons as the head sways side to side (Piraino et al. 2011). The side closest to the light (blue arrows) will receive more activation than the other side. The larva turns to and direct its crawling along an increasing light gradient, using the vector in which the average stimulation on both sides becomes equal (color figure online)

Eyeless sea urchin larvae show a photophobic response to bright light via reversal of swimming direction. The larval arms contain non-neuronal cells expressing Opsin2, an echinoderm-specific opsin that does not fit into other major opsin families (D’Aniello et al. 2015). These mesenchymal cells are thought to promote cholinergic signalling in adjacent neurons, which maintains ciliary beating and swimming in a forward direction. In the presence of very bright light this pathway is inhibited by Opsin2, which results in a ciliary reversal of the swimming direction (Yaguchi et al. 2022). This means that larvae entering the harmful strong sunlight of surface waters will swim backwards and return to a dimmer position again (Fig. 1a). Thus, a simple method of positioning oneself in optimal light environments without eyes or photoreceptors associated with pigments or membrane elaborations can be achieved.

Light can also be directly dangerous to an individual, particularly high-energy wavelengths of the ultraviolet radiation (UVR) part of the spectrum, which causes DNA damage (Malloy et al. 1997) and oxidative stress to living cells (Jager et al. 2017). Organisms that must spend their life in strong sunlight tend to have protection from the harmful radiation, employing hair, scales, thick cuticle or skin pigmentation to absorb or reflect UVR before it enters the body. They must constantly repair UVR-induced damages to survive, which has metabolic costs (Rautio and Tartarotti 2010; Stábile et al. 2021). Many marine zooplankton feed on photosynthetic phytoplankton in surface waters. They tend to have small transparent bodies that offer little protection from harmful solar radiation because protective pigmentation increases the risk from visual predation (Bashevkin et al. 2020). Therefore, UVR avoidance behaviours are very common among zooplankton. Unlike blue light, which penetrates deepest in aquatic environments, UVR wavelengths are attenuated by water more readily, so only the first 10 m of coastal surface water or < 50 m in the clearest open oceans are suffused by UVR (Mascarenhas and Keck 2018). Consequently, many animals avoid UVR by undergoing a diel vertical migration, diving down to deep water at dawn to escape both predators and harmful UVR of sunlight, then swimming towards surface at dusk to feed safely (Brierley 2014; Leach et al. 2015). This migratory behaviour has been observed among zooplankton in clear glacial lakes too, which lack threat from fish-predators; therefore UVR appears to be the main driver of community distributions there (Williamson et al. 2001).

Non-migratory and shallow water-dwelling zooplankton must stay safe from UVR in sunlit waters. As a larva, the marine bristle worm *Platynereis dumerilii* swims for several days freely in the plankton of its coastal water habitat while it grows and develops. At this stage, the larva has both rhabdomeric photoreceptors in the eyes and extraocular ciliary photoreceptors in the brain that are not able to sense direction (Arendt et al. 2004) and are UV sensitive (Tsukamoto et al. 2017; Verasztó et al. 2018). Neural connection mapping between the two photoreceptor types shows that the ciliary photoreceptors are presynaptic to the visual rhabdomeric light pathway, which is associated with its positive phototaxis (light-seeking) behaviour. Therefore, high UVR inhibits swimming towards a light source and instead, an upright larva will flip itself upside down in the water, diving downwards to escape the UVR. Even if the UVR light is presented from the bottom, this downward diving behaviour persists as the ciliary photoreceptors are unable to sense light direction and trigger downward swimming regardless of UVR direction (Gühmann 2017). Mutants with defunct UV sensing (*c-opsin1* knockouts) fail to dive in the presence of UV light (Verasztó et al. 2018). This hierarchical interaction of the two photoreceptor circuits retains the bristle worm larva in a photic environment based on the relative intensities of UVR and blue/cyan light, forming a depth gauge.

On land too, the microscopic roundworm *Caenorhabditis elegans* spends life underground in dark soil and lacks any ocular structure. Accidental exposure to bright sunlight above the soil surface is harmful to *C. elegans;* so it has a photophobic response to UVR. Movement is accelerated in the opposite direction from the region of the body that is stimulated (Ward et al. 2008). Surprisingly, the worm lacks representatives from opsin and cryptochrome protein families that mediate phototransduction pathways in other metazoans. Instead, it has a unique transmembrane receptor called LITE-1 (Edwards et al. 2008; Gong et al. 2016). This is a modified taste receptor that is sensitive to short-wavelength light, but unlike opsins, LITE-1 lacks the accessory light-sensitive chromophore. The protein can efficiently absorb UV light (Gong et al. 2016), albeit in a wavelength region where there is hardly a photon in the natural environment.

Extraocular UV avoidance (photophobia) is widespread among animals due to the universally damaging effect of the high-energy wavelengths on living cells, e.g. Birkholz and Beane (2017). Genes for photodamage stress, repair and avoidance responses appear to be highly conserved and ancient, possibly providing the original basis and molecular pathways for evolution of visual systems (Swafford and Oakley 2019).

### Scotophobia

The opposite to photopobia, the aversion to darkness or a dimming in light, is known as scotophobia. It is also referred to by some biologists as “step-down photophobia” (e.g. (Diehn et al. 1977; Sakai et al. 2020)). While not all reactions are fast, often it presents as a sudden startle response to passing shadows or looming objects. Scotophobic responses are useful to mediate escape responses from approaching predators for animals that cannot resolve the visual scene. A sudden dimming of the environment may be enough to tell the animal that a threat is looming. Likewise, animals that prefer sunlit environments can use scotophobic responses to avoid venturing further into darkened places.

An iconic example of a shadow response is displayed by adult barnacles that rapidly withdraw their cirri in response to shading. The reaction in these sessile animals is mediated by small pigmented or non-pigmented ocelli (depending on the species). The neuron types and responses involved have been described in a series of classic papers by Gwilliam (Gwilliam 1963, 1965, 1976; Gwilliam and Stuart 1990).

On detection of a shadow or sudden darkening of the light environment, sessile fan worms (Sabellidae) and Christmas tree worms (Serpulidae) rapidly withdraw their conspicuous outstretched radiolar tentacles inside their secreted tube (Nilsson 1994). Their cerebral eyes are hidden inside the tube and are of little use, so they have produced alternative arrays of radiolar photoreceptors on their delicate branchial-feeding tentacles. A vast diversity of these optical devices exist between sabellid genera (Bok et al. 2016). The radiolar tentacles of some are covered with randomly spaced and dissipated single ocelli, while others are more regularly distributed or in paired sets. The most sophisticated extraocular eyes were described in *Acromegalomma vesiculosum*. This species has a pair of radiolar compound eyes with hundreds of facets (Bok et al. 2019). The lifestyle and scotophobic tentacle-withdrawing response is almost universal among sabellids, so it is unclear why so much diversity exists in their photoreceptor types, opsins and optical solutions (lenses, pigment cells etc.) (Bok et al. 2016).

A quite different but broadly related phenomenon is represented in adult annelids. Many annelid worms have cerebral eyes but some have evolved additional light-sensing systems on other parts of the elongated body to protect them from predators, via a shadow response. As an adult worm, *Platynereis dumerilii* adopts a benthic life, protected by its secreted tube on the substrate. Long appendages called cirri protrude from the head. The cirri express Go-opsin1, which mediates a sudden aversive shortening of the body to withdraw from potential danger in response to a decrease in light intensity that may be caused by a shadow or looming object (Ayers et al. 2018).

Swimming animals can also respond to a dimming of light intensity. Planula larvae of corals are covered all over in cilia for motility (Poon et al. 2022). Lacking specialized eye structures or a centralized nervous system, the larvae swim in helices searching for suitable adult habitat within the photic zone of coastal waters. Once found, the larva will attach itself, settle and metamorphose into the first polyp of a new colony. Various sensory cells inform the planula of the presence of desirable or undesirable conditions for life as a sessile adult. It is essential that they have access to enough light for their algal symbionts to supplement their diet via photosynthesis. Dangerously shallow and turbulent water contains a lot of solar UVR, which can cause DNA damage and oxidative stress (Lesser 1997). The larvae of *Acropora* produce a delayed scotophobic response (or as the authors call it, step-down photophobia) by reducing their swimming speed a few seconds after a sudden decrease in light intensity (Sakai et al. 2020). The change in swimming speed is brought about by a ciliary arrest and a muscular contraction from bullet shaped to a shorter and more spherical profile (EB, unpublished results). If the dimmer light persists, the larva will eventually elongate and resume its usual velocity of swimming and exploration. It is thought that when the larvae encounter thresholds of suitable ambient light environments, this behaviour prevents the larvae from swimming further into dark caves, under overhangs or from venturing into deep water where light levels are unsuitable for coral colonies. Thus, the larvae have an effective non-visual light-sensing mechanism to stay within the photic zone. Historically, coral larvae have been described as phototactic, but recent use of this term for this non-directional response has been rightly avoided (Mulla et al. 2021) (see discussion for explanation).

The tadpole larva of the ascidian *Ciona intestinalis* (a sea squirt) has a simple central nervous system consisting of a small brain vesicle and a nerve chord. The neural connectome of a *Ciona* larva has been reconstructed and revealed the connectivity of the simple eyes (Ryan et al. 2016). There are two distinct types of ciliary photoreceptors in the *Ciona* larval eye (Horie et al. 2008). The outer segments of the first glutamatergic type are cupped by a pigment cell, allowing directional light sensing and negative phototaxis. The second GABAergic photoreceptor type has no pigment association and mediates a shadow response, or scotophobia: a burst of swimming in response to light dimming (Zega et al. 2006; Salas et al. 2018; Kourakis et al. 2019). The whole photosensory system consists of a handful of cells and the entire nervous system connectome is only 177 cells (Ryan et al. 2016). *Ciona*'s schotophobic response is controlled by a simple antagonistic neural pathway involving the photoreceptors in the ocellus, a gravitaxis-mediating otolith and muscles in the left and right sides of its tail to effect a sudden upward turn (when facing downwards), along with its upward swimming response (Bostwick et al. 2020). Light activation inhibits output from the otolith, however, when the light is dimmed and the inhibitory interneurons from the photoreceptors are not activated, larval reorientation and upward swimming can take effect via the now unsuppressed otolith circuit.

Shadow responses are also seen in some vertebrates. Tadpole larvae of *Xenopus* frogs spend the vast majority of their time hanging from a mucus strand attached to the surface (Roberts et al. 2000). They are known to avoid very bright, blue- and UV-rich light environments, which can increase their predation risk, preferring green-spectrum environments indicative of slightly deeper and safer water. Unattached animals actively swim to search for their preferred refuge, the shaded underside of floating objects in a pond. At this time, tadpoles reliably respond to a decrease in light intensity by turning to swim directly upwards towards the water surface (Fig. 1b). This response is controlled by the pineal eye and is thought to facilitate location of shadow-casting objects above them that they may like to attach to (Foster and Roberts 1982; Jamieson and Roberts 2000). This means that of course, they are not technically photophobes, despite showing a striking response to dimmed light. *Xenopus* larvae also retain additional non-visual opsin and cryptochrome photoreceptors in the deep-brain caudal diencephalon that can activate swimming behaviour in response to UV light, independent of eyes or pineal gland (Currie et al. 2016).

The ciliary photoreceptors of frog tadpoles and ascidian larvae are considered homologous. A scotophobic response mediated by the ancestral pineal “eye” could perhaps have been present in the last common ancestor of vertebrates and tunicates. Indeed, blind populations of the cave fish *Astyanax mexicanus* undergo regressive developmental degeneration of the lateral eyes and visual system due to total lack of environmental light during the past 1 million years or so. However, photosensitivity has been retained in the larval pineal eye during the first few days of its development (Yoshizawa and Jeffery 2008). The larvae exhibit a scotophobic shadow response similar to sighted conspecifics living in illuminated surface waters, responding to a sudden decrease in light intensity by swimming upwards towards the water surface.

### Photokinesis

Photokinesis entails modulation of swimming speed or turning frequency of an individual to match it to the ambient light intensity (Diehn et al. 1977; Nultsch and Häder 1988). The matched behavioural state remains constant and does adapt or habituate over ecologically relevant timescales, i.e. the time that the organism would take to scan an environmental light gradient.

Photokinesis has mostly been reported in bacteria employing non-opsin mediated forms of photic sensing where light capture tends to directly power locomotion. For example, many marine bacteria express proteorhodopsin, a proton pump that is light sensitive due to retinal incorporation. The bacteria use these ion pumps to absorb and harvest light for supplemental energy production as an alternative to chlorophyll-based autotrophy (Béjà et al. 2000; Olson et al. 2018). Artificial expression of proteorhodopsin in *Escherichia coli* cells can cause photokinesis whereby light is used to directly power the flagella motors, working much like a solar panel (Walter et al. 2007). Swimming speed is increased under high-intensity light so the cells formed dense accumulations of individuals in brightly lit regions and sparsely distributed in darker areas (Frangipane et al. 2018).

Ciliates can also show a photokinetic response characterized by increased ciliary beat frequency and a lengthening of the body under strong light leading to faster swimming (Matsuoka 1983; Kim et al. 1984). An extraordinary example of photokinesis exists in colonies of *Choanoeca flexa*, a rockpool choanoflagellate. Individual cells attach side by side at their microvillar collars to form a single curved bowl-shape layer. In the dark, the cell bodies line the inside, and the cilia point outwards along the convex external surface of the bowl, which allows them to beat effectively, propelling the colony through the water. Once they reach a region of bright illumination, the whole colony turns inside out, inverting to bring the cilia inside the bowl, while the cell bodies now face out. This orientation means effective ciliary swimming is impeded, causing them to keep within a well lit location. Meanwhile their cell body points outwards and flows are optimized to facilitate feeding (Brunet et al. 2019). For an extensive review of the evolution of protist photobehaviour the interested reader is referred to Jékely (2009).

**Fig. 2:**
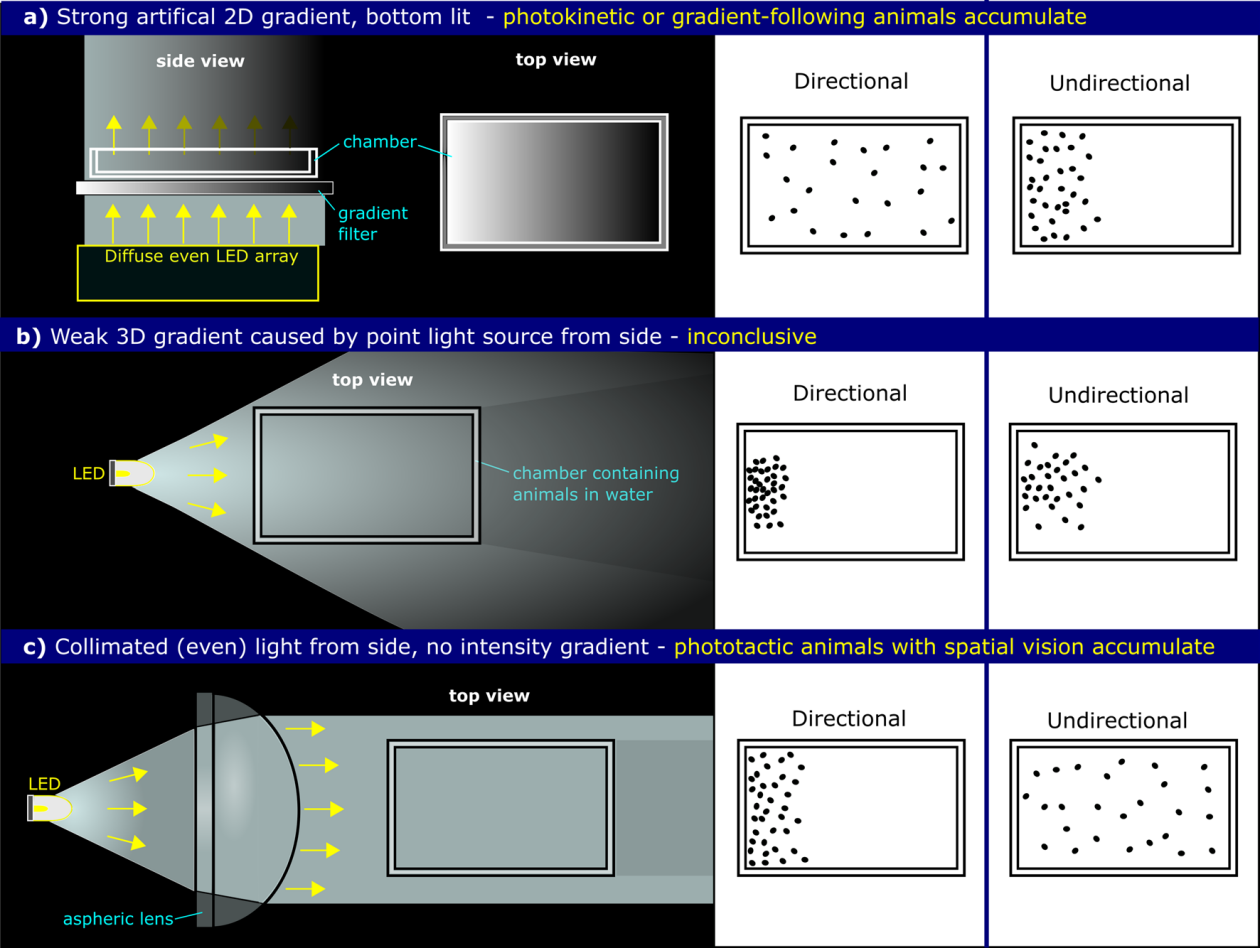
Simplified behavioural assays (left) are depicted as viewed from above a shallow rectangular dish of water containing small motile aquatic animals. The apparatuses are designed to be used in ambient darkness with specified light stimuli that allow discrimination between different photobehaviours. On the right, light-seeking animals in the dish are represented by black dots and show the hypothetical distributions that would result from time spent exposed to the light stimuli provided. These assays can help to assess whether an organism has directional light-sensing capability or not. **a** One suggested method to confirm photokinetic or intensity gradient-following photobehaviour. A LED array provides even diffuse illumination from below, which is passed through a 2D linear gradient filter (or stepped neutral density gradient). Light-seeking animals that moderate swimming speed to light intensity or compare intensity over time as they travel through space, will accumulate on one side, whereas, an animal that “sees” light direction or images will not. **b** A point light source on one side produces a weak light gradient in all directions from the source, which photokinetic animals without directional light sensors can potentially follow to show a phototaxis-like response. Positively phototactic animals with directional sensors will accumulate close to the brightest spot. This design is often used experimentally assess light-seeking behaviour, but is unsuitable to verify true phototaxis. **c** To confirm directional light sensing or spatial vision and true phototaxis, a light stimulus must be collimated into a parallel beam using an aspheric lens, to illuminate the entire chamber evenly. This will remove intensity gradients and prevent photokinetic or gradient followers from accumulating on one side (color figure online)

Photokinesis is often confused with directional phototaxis, as both responses can lead to the accumulation of organisms in brighter areas in a strong light gradient. However, the mechanisms are very different and it is important to properly distinguish them. We suggest two possible experiments to rigorously test for the photokinetic ability of an organism. In one assay, one should provide a linear light intensity gradient in space within a test chamber without a directional light cue (e.g. light projection from below while testing horizontal swimming) and detect any changes in the distribution of the organisms along the gradient (Fig. 2a). One should minimize reflections and scatter in the setup. An alternative assay would be to illuminate a test chamber evenly from below (e.g. using a diffuse LED array to eliminate directional cues) and gradually change the intensity of the illumination in time while measuring swimming speed or other aspects of behaviour (e.g. turning frequency). In both experiments, the rate of light intensity change could be varied and matched to the size and speed of the organism. If the organisms have a photokinetic ability, they are expected to accumulate in a certain zone of a spatial intensity gradient and change their speed as a function of intensity. A purely phototactic organism with direction-sensing eyespots would not be able to read out these non-directional cues (see also Discussion).

In animals, examples of true photokinesis have not been reliably demonstrated and is perhaps rarely investigated systematically. Responses such as ciliary beat frequency modulation to ambient brightness could be fairly common, but perhaps overlooked in favour of other more obvious photoresponses. One potential example of a photokinetic animal is the (also scotophobic) coral larva *Acropora* sp., where the amplitude of swimming speed reduction is roughly proportional to the change in intensity of ambient light experienced (Sakai et al. 2020) (Fig. 1c).

### Phototaxis

Phototaxis is an organism’s movement towards (positive) or away from (negative) a directional light source. Phototaxis does not rely on a light intensity gradient and is distinguished by directional light sensing (Fig. 2b). There are two main sensory-motor mechanisms of phototaxis, visual and non-visual. The two are separated by whether or not body movement (e.g. rotation or head casting) is required for directional light sensing (Nilsson 2009; Randel and Jékely 2016). Optical mechanisms and evolutionary origins of phototaxis have been reviewed previously (Jékely 2009; Randel and Jékely 2016), so to avoid exhausting the topic, we will focus here on non-visual phototaxis, in particular some rather unusual extraocular strategies that appear to function without conventional pigment shading.

The conventional forms of non-visual phototaxis rely on photoreceptor cells that are partially shaded by pigment cells. Spatial discrimination is provided by shading combined with helical rotation or side-to-side head casting. This strategy measures changes in light intensity over time (rather than in space as in visual phototaxis) and some form of a scanning motion is an essential component of sensing (Jékely 2009; Nilsson 2009; Randel and Jékely 2016). Thus, indirect light direction sensing and phototaxis can be achieved without “seeing” the light source itself like spatially resolving eyes can (McHenry and Strother 2003; Nordström et al. 2003; Jékely et al. 2008). However, pigment shading is not the only way to endow the organism with the ability to detect light direction. There are other unusual forms of directional discrimination, including refractive light focusing and body shading. Below we discuss examples of these unconventional mechanisms of optical discrimination.

In the cyanobacterium *Synechocystis,* the whole cell itself acts as a tiny spherical lens to focus light on to the far side of the cell membrane with respect to light direction (Schuergers et al. 2016). The membrane contains photoreceptor molecules all over, but those in the region where light is focused are activated maximally, causing the cell’s pili to move the cell towards the light source on a surface.

Freely swimming volvocine algae (Kessler et al. 2015) and *Chlamydomonas* have eyespots that employ a conventional non-visual strategy with shading pigment. Nevertheless, *Chlamydomonas* mutants that lack the carotenoid screening pigment in the eyespot can still respond to directional light cues and show phototaxis. In this case, spatial discrimination during helical swimming is ensured by the lens effect of the convex cell body, but the sign of phototaxis is reversed due to lack of screening pigment reflection (Fig. 1d). Directional light can be focused in a particular orientation on the region of the plasma membrane containing the photoreceptor molecules (Ueki et al. 2016).

A curious form of scanning phototaxis is present in the multicellular slug stage of the slime mould *Dictyostelium discoideum*. *Dictyostelium* begins life as a spore, germinating into many independent unicellular amoebae. These cells gather and join together to form a multicellular slug, a slime-covered bag of amoebae that can glide along the soil substrate. Eventually, when a suitable habitat is reached, the slug consolidates into a ball that begins to form fruiting bodies (Bonner 1944; Gaudet et al. 2008). At the slug stage, although the individual cells have no method of detecting spatial information from light, the whole slug is positively phototactic (Marée et al. 1999). The mechanism works via light-dependent chemotaxis. Directional light is focused on the nearside of the slug, inducing cAMP release in those cells and a chemical concentration gradient across the body. The cAMP wave propagates through the slug to chemosensitive receptors, which activate locomotion in the direction of the cells closest to the light source (Marée et al. 1999; Miura and Siegert 2000).

*Schistosoma mansoni*, the trematode blood fluke, is a parasitic flatworm with a complex life cycle, ultimately infecting a mammalian host. The freshly hatched ciliated miracidium stage swims in helices through freshwater bodies to locate its first amphibious snail host, which tends to stay close to the water surface. Miracidia also accumulate at the surface of a beaker of water if light is shone down from above. The larvae show effective positive phototaxis to directional light, so shining a collimated beam of light up into a container of *Schistosoma* miracidia from below causes downward swimming (observations by EB, GJ and K. Rawlinson), which excludes gravitaxis or intensity gradient-following photokinetic mechanisms. The larvae have no pigment shading in the region of the r-opsin-expressing photoreceptor cells that flank the brain. In contrast, their close relatives, the liver fluke *Fasciola hepatica,* do have pigmented rhabdomeric eyespots to mediate phototaxis (Mattes 1949; Isseroff and Cable 1968; Wilson 1970). *S. mansoni* larvae thus seem to have lost the ancestral shading pigment and may instead rely on bodily focusing to concentrate light on to the photoreceptor cells located on either side of the body, providing some directional discrimination. This strategy may allow the larvae to remain transparent and better evade visual predators.

The *Drosophila* larva achieves negative phototaxis by sweeping its head from left to right as it crawls, scanning for decreasing light intensity to inform turning. The two larval eyes on the left and right side of the head are unable to obtain directional light information alone, but as a pair, the signals are compared to provide a two-pixel view of the world (Kane et al. 2013; Humberg et al. 2018).

The hydrozoans *Clava multicornis* and *Hydractinia echinata* have an elongated planula stage with a larval nervous system. These planulae crawl along surfaces on their cilia and show non-visual scanning phototaxis, moving towards a light source (Katsukura et al. 2004; Pennati et al. 2013). As the larva crawls, swinging its aboral pole from side to side, it is able to compare light intensity between the two sides, obtaining some directional information. The *C. multicornis* nervous system has the typical polarity along the anterior-posterioir (aboral-oral) axis that is common with planulae, whereby the aboral end contains a nerve plexus. However, neuropeptide staining has revealed an array of RF-amide-immunoreactive presumptive sensory cells that are largely clustered into two clumps on either side of the anterior pole (Piraino et al. 2011). These clusters of RFamide-expressing cells are prime candidates as photoreceptor cells mediating phototaxis (Fig. 1e). *Hydractinia* has similar neuropeptides and appears to exhibit the same head-swinging scanning behaviour during its phototaxis. In *Hydractinia*, exogenous RFamide application inhibits phototaxis, suggesting that neuropeptide release and peptidergic transmission from the sensory cells may regulate the response (Katsukura et al. 2004).

In the flatworm *Schmidtea mediterranea*, peripheral extraocular rhabdomeric photoreceptors mediate negative phototaxis away from UV light (Shettigar et al. 2021). These photoreceptors line the periphery of the animal’s body and are not associated with pigment. Directional sensitivity is likely conferred by body shading.

## Distributed visual systems

Distributed individual extraocular photoreceptors on a body that point in different directions provide an alternative mechanism for enabling spatial light sensing. These distributed photosensory systems are common among molluscs (Nilsson 1994; Land 2003; Speiser et al. 2011) and echinoderms (Ullrich-Lüter et al. 2011; Garm and Nilsson 2014; Sumner-Rooney et al. 2018, 2020).

The sea urchin *Diadema africanum* has a crude spatial vision without eyes. The round body itself shades light from photoreceptors on the tube feet from one side, allowing directional sensing. The urchins can resolve objects spanning 29°–69° in their visual scene and use this low-resolution vision to move towards darker areas. They also show a defensive startle response to a looming stimulus, pointing their spines towards the object (Kirwan et al. 2018).

Adults of the sea anemone *Exaiptasia *sp live as solitary polyps and lack ocular structures. They can shuffle along on their muscular foot, albeit slowly, to relocate to a more desirable environment. Like corals, they contain symbiotic dinoflagellates (zooxanthellae), which photosynthesize and supply them with nutrients (Glider et al. 1980; Garrett et al. 2013). Therefore, they prefer environments with plenty of light and are known to crawl along a light gradient or bend their zooxanthellate-rich tentacles towards a light source (phototropism) (Foo et al. 2020). Recently, aposymbiotic polyps (lacking zooxanthellae) have been shown to lack directionality in movement and while they do actively move around, they appear unable to detect the light source, failing to settle in brightly lit areas, unlike equivalent polyps that do possess symbionts (Foo et al. 2020; Strumpen et al. 2022). Possible mechanisms include the local sensing of photosynthetic activities such as oxygen (Strumpen et al. 2022) or body shading provided by the pigmented symbionts leading to stronger activation of *Exaiptasia* opsins on the illuminated side. The interpretation of the available data is complicated by differences in opsin expression profiles between symbiotic and aposymbiotic host anemones (Gornik et al. 2021).

Another group of organisms where distributed photoreceptors could provide directional or spatial information is fan worms. Many species have structures more complex than would be required for a simple shadow response (discussed above) (Bok et al. 2016). Their retraction response to shadows does not require spatial information as the worm only retracts away in one direction (inside the tube), regardless of direction of the threat. However, many sabellid species have hundreds of eye spots. These distributed detectors may allow the broad pooling of signals across the tentacle field, enabling noise filtering to prevent false alarms (Bok et al. 2019). Alternatively, the repetitive structures may serve as backups in the event of partial tentacle loss. An exciting further possibility is that the different tentacles can integrate spatial information to provide a crude image of the environment. This, however, would require relatively advanced neural cognition.

While some chitons are eyeless, the hard shell plates of some species (e.g. *Acanthopleura granulata)* are embedded with small lensed image-forming eyes that enable shadow detection (dimming in parts of the visual scene) and defensive contractions of the foot to prevent dislodgement (Speiser et al. 2011). However, there are other genera (e.g. *Chiton *sp.) with smaller and more numerous simple light-sensitive structures that are paired with sensory organs called aesthetes. These pigmented simple “eyes” individually lack the ability to form images, but together they appear to work as a network to provide spatial information on the scene (Kingston et al. 2018). Behavioural tests show that although they do not respond to shadows, they can resolve and sometimes move towards darker objects as small as 10 degrees using this distributed visual system. For further reading, a comprehensive review on the subject of distributed vision has recently been published (Buschbeck and Bok 2023).

## Light quality

### Wavelength discrimination

The amino acid sequence of an opsin and its specific way of bonding to a retinal chromophore determine the peak wavelength and range of the electromagnetic spectrum that this visual pigment is excited by (Terakita 2005). Water attenuates some parts of the light spectrum more readily than others and many aquatic animals live at depths where little red (long-wavelength) light penetrates. Animals tend to have photopigments with spectral sensitivity tuned to match the light in their environment. Red light is filtered out in just a few metres of seawater, while blue/cyan wavelengths (~ 480 nm) penetrate deepest. Therefore, marine animals are mostly sensitive to blue/cyan light and lack long-wave (red) receptors (Warrant and Johnsen 2013). The exceptions are those that live in coastal or shallow water habitats.

With a single photopigment type, it is possible to detect light intensity of wavelengths in a certain excitation range, but no information on spectral composition can be learned from only one channel. Colour vision is often associated with sophisticated visual systems to, e.g. increase visual contrast. However, the task of wavelength discrimination can hypothetically be achieved by any animal and all that is needed is two or more spectral classes of photoreceptors and some downstream signal processing to enable neural comparisons. This ability can be very useful to assess the spectral composition of environmental light. In small aquatic animals lacking spatially resolved vision, comparing excitation signals from long- and short-wave regions of the light spectrum could be used to assess depth, time of day or sediment loading in the water, all of which can produce characteristic colour spectra.

Assessing whether the ambient light is rich in UVR wavelengths can help protect an animal from harmful radiation. In some cases, extraocular UVR sensing is integrated with blue/cyan light sensing in the cerebral eyes. The *Platynereis* larva switches its behaviour from positive phototaxis (light seeking) under low UVR to downwards diving under high UVR (Verasztó et al. 2018). On land, *C. elegans* lacks eyes or opsins, but colour discrimination behaviours have been demonstrated (Ghosh et al. 2021). The worm’s chosen habitats tend to be nutrient-rich compost soils with absorbent pigments that create a characteristic environmental colour spectrum (amber). Strong white or blue light can indicate departure from this safe environment and risk of exposure to harmful UVR or predators. The worms appear to assess ratios of blue and amber light employing the blue-sensitive LITE-1 protein and other unknown sensors (Ghosh et al. 2021).

The sea anemone *Anthopleura xanthogrammica* shows at least three different wavelength-dependent photobehaviours. Dark-adapted adults display tentacle retraction to UVB radiation (peak at 280 nm), while oral disk flexion and vertical contraction of the column occur under UVA–blue light (peak at 360 nm). Thirdly, tentacle flexion responses peak at both 360 nm and under 500 nm visible light (Clark and Kimeldorf 1971). This suggests the presence of three distinct opsins and extraocular photosystems tuned to respond to different qualities of light.

The ciliated larvae of the sponge *Amphimedon queenslandica* (formerly *Reniera *sp.) show two peaks of sensitivity along the light spectrum (near 440 nm and 600 nm) (Leys et al. 2002). However, the sponge does not produce opsins and has only cryptochromes for photopigments, so it is unclear how the long-wavelength sensitivity is achieved and what its ecological relevance is.

In *Acropora* coral larvae, wavelength discrimination may be important in their habitat choice. The crustose coralline algae that the larvae prefer to attach to and settle on strongly reflect pink-red light that is visible in their shallow water habitat. The larvae express multiple acropsins (at least six types in *A. millepora*) (Mason et al. 2023) and electrophysiology revealed that an acroporid (and several other coral species) have distinct sensitivity to long-wavelength (red) light, in addition to blue sensitivity (Mason et al. 2012). Although red light does not appear to induce active swimming (Sakai et al. 2020), there is some evidence to suggest that larvae also prefer to settle on substrate that reflects red light, at least under artificial conditions (Mason et al. 2011; Strader et al. 2015; Foster and Gilmour 2016; Ricardo et al. 2021). Whether blue- and red-light sensitivity are used in different tasks (i.e. swimming behaviour vs. settlement decisions), or the coral larva is able to integrate information from different spectral channels that feed into the same behaviour is currently unknown and there is no affirming evidence yet for a single species.

### Polarized light sensing

Polarization sensitivity is the ability to discriminate the electric field vector (e-vector) component of light (Heinze 2013; Foster et al. 2018). There are many polarized light sources in nature. The sky has a distinctive polarization pattern caused by Rayleigh scattering that is near-invisible to us but highly conspicuous to many animals (Horváth et al. 2014) and is used by many flying insects for orientation and flight stability (Heinze 2013). Reflections from flat shiny surfaces are rich in polarized light that can be used as a cue for polarotaxis to locate objects like water bodies, leaves or bodily surfaces of other individuals (Wehner 2001; Foster et al. 2018). While this property of light goes unnoticed by humans, alignment of photoreceptor cell membranes and the opsins embedded within them in a single plane will let an animal’s eye collect light with just the matching e-vector of light, creating a polarization filter. Adding a second set of photoreceptors with membranes and opsins in the orthogonal orientation provides the architecture needed to discriminate e-vectors. For example, such membrane arrangement occurs in the highly ordered photoreceptors in crustacean eye ommatidia (Marshall and Cronin 2014).

Polarization sensitivity is commonly presented in complex or simple eyes, but there are a few examples of extraocular photoreceptors within distributed visual systems that employ polarization filters. The first known example was a brittle star that uses endoskeletal ossicles as polarizing filters and is able to discriminate polarized from unpolarized light (Johnsen 1994). It is thought that the detection of strongly polarized light cues is an essential proxy for indicating high UVR levels as part of their shade-seeking behaviour (Johnsen and Kier 1999). Recently, a chiton has also been shown to filter polarized light using its birefringent aragonitic lenses (Chappell and Speiser 2023). In vertebrates too, lizards can detect the polarized sky compass with ordered photoreceptor membranes in their parietal “third” eye (Hamasaki and Eder 1977; Beltrami et al. 2010).

## Discussion

We have illustrated only a few notable examples of animals that have evolved effective non-visual or extraocular solutions for detecting light to inform locomotive behavioural photoresponses (listed in Table 1). The cell types involved, the optical structures and effector mechanisms tend to be as diverse as the animals themselves. Extraocular photoreception mechanisms and signalling can be comparatively more challenging to understand or even identify, compared with eyes. Many systems have been historically overlooked due to inconspicuous photoreceptor morphology lacking distinctive modified cell membrane or lack of accessories such as pigment cells. Recently, however, increased use of exploratory molecular techniques have revealed a greater diversity and prevalence of photoreception types than previously thought (Porter 2016).

**Table 1:** Examples of the photosensory solutions that animals employ to guide photobehaviours

Behaviour	Light avoidance and shade seeking	Dark avoidance and light seeking
*Non-directional light sensing*		
Muscular body retraction or contraction	*Anthopleura xanthogrammica* (Clark and Kimeldorf 1971)	*Platynereis dumerilii* (ragworm) adults (Ayers et al. 2018) *Sabella *sp. (fan worms) (Nilsson 1994) and serpulids (Christmas tree worms) (Bok et al. 2017) *Chiton tuberculatus and C. marmoratus* shadow response (Kingston et al. 2018)
Stop or pause swimming		*Acropora tenuis* (coral) larva (Sakai et al. 2020)
Direction reversal	Echinoid (sea urchin) larva (Yaguchi et al. 2022) *Blepharisma japonicum* (protozoan) (Matsuoka 1983)	
Burst of movement	*Drosophila melanogaster* (fruit fly) larva	*Ciona intenstinalis* (Zega et al. 2006)
Swimming up or down	*Platynereis dumerilii* (ragworm) larva UVR avoidance (Verasztó et al. 2018) *Porites astreoides* (coral) larva UVR avoidance (Gleason et al. 2006)	*Astyanax mexicanus* (cave fish) larva pineal eye (Yoshizawa and Jeffery 2008) *Xenopus laevis* (frog) tadpole larvae pineal eye (Foster and Roberts 1982; Jamieson and Roberts 2000)
*Directional light sensing*		
Phototaxis using distributed visual systems	*Diadema africanum *and* Strongylocentrotus purpuratus* (sea urchins) tube feet (Ullrich-Lüter et al. 2011; Kirwan et al. 2018) *Chiton *sp. (Kingston et al. 2018) *Ophiocoma wendtii* (brittle star) (Sumner-Rooney et al. 2018, 2020)	*Argopecten irradians* (bay scallop) (Chappell et al. 2021)
Movement towards/away from a stimulated body region	*Schmidtea mediterranea* (flatworm) extraocular UVR sensing and avoidance (Shettigar et al. 2017) *Caenorhabditis elegans (*roundworm) (Ward et al. 2008)	*Exaiptasia pallida* (sea anemone) with symbionts (Foo et al. 2020) *Dictyostelium discoideum* (slime mould) (Marée et al. 1999; Miura and Siegert 2000)
Scanning phototaxis (without pigment)	*Drosophila melanogaster* (fruit fly) larval eyes and head sweeping (Kane et al. 2013; Humberg et al. 2018)	*Clava multicornis* and *Hydractinia echinata* (Katsukura et al. 2004; Piraino et al. 2011; Pennati et al. 2013) *Schistosoma mansoni* (blood fluke) miracadia (Mason and Fripp 1977)
Defensive movement	*Tridacna maxima* (giant clam) mantle eyes (Land 2003)	*Sabella *sp. (fan worms) (Nilsson 1994) and serpulids (Christmas tree worms) (Bok et al. 2017) *Arcidae* (ark clams) (Nilsson 1994) *Acanthopleura granulata* (chiton) (Speiser et al. 2011)
*Light quality sensing*		
Wavelength discrimination	*Anthopleura xanthogrammica* (sea anemone) (Clark and Kimeldorf 1971) *Platynereis dumerilii* (ragworm) larval depth gauge (Verasztó et al. 2018) *Caenorhabditis elegans (*roundworm) (Ghosh et al. 2021)	*Acropora tenuis* (coral) larva (Mason et al. 2011; Strader et al. 2015; Foster and Gilmour 2016; Ricardo et al. 2021)
Polarized light	*Ophioderma brevispinum* (brittle star) ossicles (Johnsen 1994; Johnsen and Kier 1999)	*Acanthopleura granulata* (chion) aragonitic lens (Chappell and Speiser 2023) *Podarcis sicula* (ruin lizards) ordered photoreceptors in Parietal eye (Beltrami et al. 2010)

Light-guided behaviours are varied too and can sometimes be poorly assessed or misconstrued. The ability to detect light direction is not necessary for photophobic, scotophobic, photokinetic or non-visual phototactic responses. The system must simply compare light intensities over space or time. Sometimes these responses are so effective at removing the individuals away from unfavourable light environments, that they can be mistaken for true visual phototaxis, so it is important to pay careful attention to the optical apparatus and behavioural mechanisms in place. Very often, behavioural experiments make use of an animal in a small arena with point light source from one side, which creates a gradient of light in all directions from it (Fig. 2b). The photoreceptors in any small animal can detect changes in light intensity in space or time as the body travels through it, even without spatial discrimination abilities. If bright light is being sought and it is getting brighter in time, it makes sense to simply stay on that trajectory, but if the light intensity is dimming you can change direction until it does begin to brighten. Using a wide collimated beam of light from one side of an arena creates an (almost) even beam of parallel waves with no intensity gradient (Fig. 2c). Photoreceptors lacking spatial or directional discrimination would struggle to discern from which direction the light comes from, as no matter where the animal swims, the light intensity stays uniform. It is possible to disentangle these responses using carefully considered behavioural assays. However, the potential for an animal to display more than one type of photobehaviour should be noted.

Taking a “Gallistelian” approach (Gallistel 1981), animal behaviours can be categorized broadly with three elementary units. First are reflexes, for example optical kinetic nystagmus responses used in gaze stabilization. Second are the oscillators, effectors that work regularly on a periodic basis. And third are servomechanisms, behavioural adjustments made to compensate or correct for a perceived error on the animal’s current course of behaviour (Cheng 2022). Many locomotive actions such as walking or swimming are controlled with rhythmic behaviours: oscillators and the input from photoreceptors can aid in navigation via servomechanism systems, a feedback loop in which the action being carried out serves to reduce the divergence from the sensory system’s ideal (Cheng 2023). An example of a reflex might be the aversive contraction responses of fan worms to passing shadows, whereas most navigational tasks like phototaxis are controlled with servomechanisms, corrections on directional course in response to changes in sensory cues such as light intensity. This is clearly shown in the non-visual scanning phototactic behaviour of hydrozoan larvae, as they swing their light-sensitive front ends to scan the scene. Some behaviours use a mixture of these and other mechanisms; for instance helical phototaxis relies on a behavioural oscillator (helical swimming) that is tuned by a servomechanism (sinusoid light input and trajectory adjustment). Overall, it is often difficult to categorize the phenomena discussed in this review under one of these categories. Furthermore, even the seemingly simplest forms of ‘reflexes’ will be richly modulated and context-dependent (e.g. circadian or hunger state).

While it is nice to observe the world with colour and acute spatial vision, the eyes needed tend to be large and very costly (Laughlin et al. 1998; Niven et al. 2007; Niven and Laughlin 2008; Okawa et al. 2008; Warrant and Dacke 2016). In an extreme example, the blowfly spends around 10% of its resting metabolic energy rate maintaining its high-functioning visual system (Laughlin et al. 1998). Therefore, although some groups took the well-known evolutionary roads to eye complexification, a huge contingent of animals did not. With lifestyles that simply do not require high-functioning visual systems, their energy can be devoted elsewhere (Land and Nilsson 2012). Numerous animals have retained slow or sessile lives, investing in strong defences to outwit or avoid their predators. Others took to a life of parasitism or hiding away under sediment. Lacking the element of pursuit to their feeding and possessing natural barriers from their own predators, these animals have lower selective pressure for high-functioning visual systems, compared with more actively motile animals (Warrant and Dacke 2016; Sumner-Rooney 2018).

Many animals simply do not have space or time to build a complex eye. Most aquatic invertebrates often have short, transient, planktonic larval stages, which make up a large part of the zooplankton (Young et al. 2002). As embryos develop to larvae, there is limited time and space on their little bodies to grow complex eyes. At this time, they are vulnerable and exposed to predators in the water, unable to swim against anything more than a weak current. Diel vertical migration through the water column is a common behaviour in marine zooplankton including larval stages with or without image-forming eyes (Ringelberg 1999; Leach et al. 2015). In addition, many eyeless animals show a shadow response to sudden decrease in light intensity. Ciliated swimming is common in very small, larval, or unicellular aquatic microorganisms and likely preceded muscular swimming, which requires specialized muscular-epithelial cells and a larger body size. Therefore, cilia may have provided the first direct effectors involved in early light responses (Jékely 2009; Yaguchi et al. 2022).

It is also likely that similar simple non-visual light-sensing structures provided the substrate for the evolution of more complex visual eyes. On a broader scale, light-sensing and eye evolution is thought to be a major driver of the “Cambrian Explosion” (Land and Nilsson 2012; Nilsson 2013), an era of huge evolutionary emergence. Some animals did take well-known evolutionary paths of eye improvement, adding optics such as expansive retinal pixel arrays, pigment shading to enable directional vision, lenses to focus light into an image on the retina. Investment in eyes and vision opened up new niches of visual ecology, producing highly specialized and acute vision for some, with impressive visual ecologies to match. Other animals, however, retained or continued to elaborate their simple light-sensing systems to efficiently guide behaviour. By focusing on mostly eyeless animals or life cycle stages here, we hope to demonstrate that there is a vast diversity of simple photosensitive structures to mediate effective photobehaviours, allowing many animals to thrive without significant investment in a visual system. It can be argued that these systems are not only more prevalent than eyes among animals, but that even animals with well-developed visual systems seem to have maintained or added extra light-sensing pathways instead of dispensing with them (e.g. parietal eye) (Eakin 1973; Arendt et al. 2004; Sexton et al. 2012; Kingston and Cronin 2016).

We tend to regard eye evolution as an inevitable progression from non-visual eyes to the most advanced visual systems, (primates, birds of prey, airborne hunting insects, cephalopods, etc.), considering simpler visual systems to be ‘primitive’ stop-offs along the way, or even suboptimal products of evolutionary pitfalls and traps. While we do not dispute there are good examples of these (Kirschfeld 1976; Muntz and Raj 1984; Land and Nilsson 2012), in most cases, animals have exactly the kind of photoreceptive structure they need to succeed and can afford. These have been subject to millions of years of evolution, just like the more sophisticated visual systems. Thus, we argue that the simple photodetection systems discussed here should also be regarded as adequately functional and successful sensory-system end products.

## Data Availability

Not applicable.
